# Listening to pulses of radiation: design of a submersible thermoacoustic sensor

**DOI:** 10.1038/s41598-020-68954-8

**Published:** 2020-07-24

**Authors:** Rafael Barmak, Geraldo Cernicchiaro

**Affiliations:** Brazilian Center for Research in Physics (CBPF), COMAN, Rio de Janeiro, 22290-180 Brazil

**Keywords:** Physics, Applied physics, Astronomy and astrophysics, Particle physics, Techniques and instrumentation

## Abstract

Nowadays, various collaborations are creating immense machines to try to track and understand the origin of high-energy cosmic particles (e.g., IceCube, ANTARES, Baikal-GVD, P-ONE). The detection mechanism of these sophisticated experiments relies mainly on an optical signal generated by the passage of charged particles on a dielectric medium (Čerenkov radiation). Unfortunately, the dim light produced by passing particles cannot travel too far until it fades away, creating the necessity to instrument large areas with short spacing between sensors. The range limitation of the optical technique has created a fertile ground for experimenting on the detection of acoustic signals generated by radiation—thermoacoustics. Despite the increased use of the thermoacoustic technique, the instrumentation to capture the faint acoustic signals is still scarce. Therefore, this work has the objective to contribute with information on the critical stages of an affordable submersible thermoacoustic sensor: namely the piezoelectric transducer and the amplifying electronics. We tested the sensor in a $170\,{\textit{l}}$ non-anechoic tank using an infrared ($\lambda =1064\,\hbox {nm}$) Q-switched Nd:YAG laser as a pulsed energy source to create the characteristic signals of the thermoacoustic phenomena. In accordance with the thermoacoustic model, a polarity inversion of the pressure signal was observed when transiting from temperatures below the point of maximum density of water to temperatures above it. Also, the amplitude of the acoustic signal displayed a linear relationship with pulse energies up to $(51.1 \pm 1.7)\,\hbox {mJ}$ ($R^2 \sim 0.98$). Despite the use of cost-effective parts and simple construction methods, the proposed sensor design is a viable instrument for experimental thermoacoustic investigations on high-energy particles.

## Introduction

In the 1950s, the generation of acoustic signals by the passage of a beam of charged particles was studied^1^, unfolding the possibility for a novel type of radiation sensor and consequently creating a new investigation field of acoustic effects generated by radiation. This new field is commonly referred to as photo/optoacoustic or, more specifically, as thermoacoustics when the thermal expansion mechanism is responsible for the acoustic emissions.

According to the thermoacoustic model^1–3^, a pulse of energy deposited in a liquid medium will incur in quasi instantaneous heating, relative to the sound speed in the liquid. The change in temperature will expand a portion of the irradiated region (or contract, depending on the value of the volumetric thermal expansion coefficient $\beta$ of the material) in an accelerated movement, forming a pressure pulse that propagates through the volume.

The investigation of the phenomena of sound generation by radiation was stimulated mainly by the advances in the field of high-energy physics^4^, and today the interest in understanding the origin of ultra-high energy cosmic rays have been consolidated, through big collaborations, in projects like the IceCube experiment in Antarctica^5^, in the underwater observatories ANTARES and KM3NeT, in the Mediterranean sea^6^, the Baikal-GVD, in Russia^7^, and the future P-ONE, in the Pacific ocean^8^.

These observatories have in common the photomultiplier tubes (PMTs) as sensors for Čerenkov radiation^9^, in the form of light produced by the passage of particles in the water, or ice. However, the high optical attenuation of these mediums^10–12^ constrains the detection volume imposing the necessity to increase the density of sensors and to instrument larger extents to enhance the chances of detection. Also, PMTs are not easily integrated in instruments. Some models can have sizes of 25 cm, or more, and also require high-voltage electronics to operate^13^.

The range limitation of the optical technique and thus the restriction on the size of the observatories have created a fertile ground for experimenting with less traditional methods. Two popular techniques are the Askaryan radiation, where a radio-frequency (RF) pulse is generated by the resulting cascade from the interaction of an ultra-high-energy neutrino^14–16^, and thermoacoustics. On both, the signal of interest can propagate long distances on dense mediums^17, 18^. Although the focus of this work is on the thermoacoustic technique, it is worth mentioning the possibility of future hybrid detectors using radio and acoustic detection. When using ice as a target, the Askaryan radiation signal has an attenuation length of approximately 1 km^19^ while the thermoacoustic signal is about 300 m^20^. But, when water is used as a detection medium, the thermoacoustic effect turns out to be more efficient^17^.

Recently, observatories around the world started to experiment with thermoacoustic detection. As examples, the SPATS (South Pole Acoustic Test Setup) experiment^21^ on the IceCube, and the AMADEUS (ANTARES Modules for the Acoustic Detection Under the Sea)^22^, are using thermoacoustic sensors^23, 24^ as an alternative detection method for cosmic neutrinos.

It is important to note that the thermoacoustic technique has been found valuable in very diverse fields, and not only in astrophysics. An example of a small subset of novel applications are spectroscopy, where the thermoacoustic signal might change depending on the composition of a substance (e.g., CO_2_ sensors^25^, oil contamination in water^26^). In Metrology/thermometry, since the thermoacoustic signal amplitude changes with the medium temperature, the target temperature can be inferred^27–29^. In Oceanography, where pulses of energy can be used to control submerged instruments^30^. And ultimately, since the sensor described here has a similar structure of a hydrophone^31^, it can also be used to study underwater acoustic phenomena.

Although the increased interest in the thermoacoustic technique, the instrumentation to acquire the faint thermoacoustic signal is still scarce and under significant development. Therefore, this work has the objective to contribute to the development of a submersible thermoacoustic sensor, with high sensitivity, low-noise, and cost-effectiveness, by reducing the necessity of sophisticated equipment or processes.

### Theoretical background

The pressure field $p(\vec {r},t)$, generated by the deposition of energy density in the medium $q(\vec {r}',t)$, despised the effects of viscosity and acoustic attenuation, is governed by the wave equation^2, 3^,

1 $$\begin{aligned} \vec {\nabla }^2 p(\vec {r},t) - \frac{1}{c_s^2} \frac{\partial ^2p(\vec {r},t)}{\partial t^2} = -\frac{\beta (T)}{C_p} \frac{\partial ^2 q(\vec {r}',t)}{\partial t^2} \end{aligned}$$

where $\vec {r}$ refers to the observer position, $\vec {r}'$ the point of energy deposition, $C_p$ the specific heat of the medium, $\beta$ is the thermal expansion coefficient of the medium and $c_s$ the velocity of the sound in the medium.

The thermal expansion coefficient $\beta$ of the water, the medium used as a target, has an anomalous behavior essential to establish if a signal has a thermoacoustic origin. When water is approximately at $4\,^{\circ }\hbox {C}$, known as the water point of maximum density, $\beta$ tends to zero. When the water temperature is above or below that point, $\beta$ will change its polarity; negative for $\hbox{T}_{\mathrm{H2}{\mathrm{O}}} < 4\,^{\circ }\hbox {C}$ and positive for $\hbox{T}_{\mathrm{H2}{\mathrm{O}}} > 4\,^{\circ }\hbox {C}$. The fact that $\beta$, and consequently the thermoacoustic signal, inverts its polarity and vanishes when close to the point of maximum density, it is a peculiarity of the thermal expansion mechanism, and decisive to experimentally identify the physical phenomena that generated the signal^32^.

## Sensor architecture

**Figure 1:**
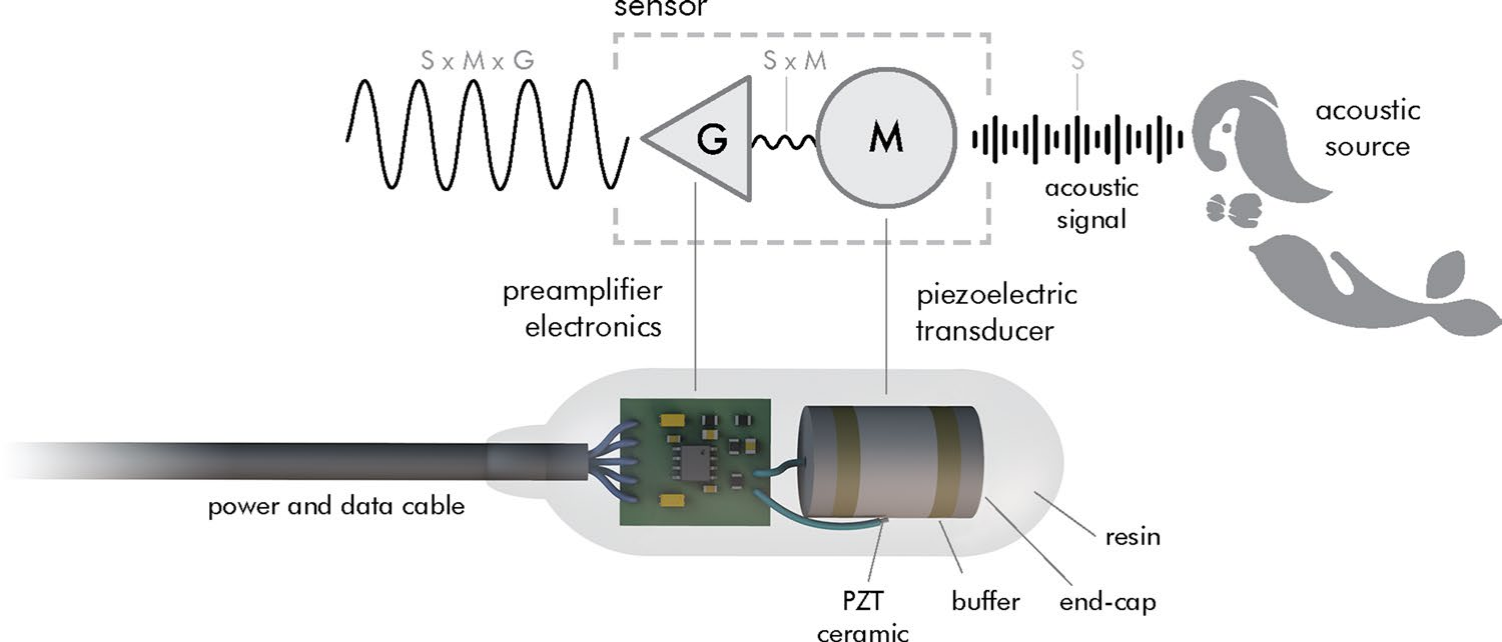
The top part displays the logical diagram of the thermoacoustic sensor signal path, where an acoustic signal is converted into an amplified electrical signal. The bottom diagram represents the idealization of the physical equivalent for the logical diagram, depicting each sensor component inside the resin encapsulation.

One method to detect the acoustic emissions generated by the interaction of radiation with a liquid is through the use of an element capable of transducing acoustic energy into electrical energy. Usually, the ability of these transducers to replicate the acoustic signal in the electrical domain is limited by its small sensitivity, $M_0$ (in V/$\upmu \hbox {Pa}$ or dB re V/$\upmu \hbox {Pa}$, demanding an amplifying stage, of gain G, so the signal can be used on following stages—e.g., filtering, digitization, processing (Fig. 1).

### Transducer

The first step in the design of a sensor is the selection of the electroacoustic transducing element. A careful selection must be made since this element will define parameters like bandwidth, sensitivity, and minimum system noise. Fortuitously, materials with piezoelectric properties are suitable for sensing low-level acoustic signals, and piezoelectric ceramics can be easily acquired in different shapes and compositions.

We opted for cylindrical elements with radial polarization and manufactured of PZT (lead zirconate titanate). The cylindrical geometry enables the construction of a device with good resistance to hydrostatic pressure, where the internal surface can be isolated from the exterior through the use of end-caps to achieve higher sensitivities^33^. The cylindrical symmetry also presents omnidirectionally on the plane perpendicular to the tube axis^34^.

#### Sensitivity

Since the efficiency of the thermoacoustic mechanism is low, on the order of $\eta = 10^{-12}$ to $10^{-18}$^35^, it is necessary to choose a transducer arrangement that maximizes the sensor sensitivity ($M_0$), improving the detection of low amplitude signals. Through the work of Langevin^33^, one can calculate the sensitivity of a shielded cylindrical transducer in function of the ratio between the internal and external diameters, $\phi = ID/OD$, and the electric potential piezoelectric constants of the ceramic compound, $g_{31}$ and $g_{33}$,

2 $$\begin{aligned} M_0 = \left| \frac{V}{p_0} \right| = \frac{OD}{2} \left[ g_{33}\left( \frac{1-\phi }{1+\phi } \right) + g_{31} \right] \,\,\, [V/\mu Pa]. \end{aligned}$$

We selected the Steminc SMC1513T10410 piezoelectric ceramic as the transducer for our sensor mainly for its low-cost ($23.00/un) and its accessibility. This ceramic has a resonant frequency of ($$65 \pm 5$$) $$\hbox {kHz}$$, and the following dimensions: $$\hbox {OD} = 15\,\hbox {mm}$$, $$\hbox {ID} = 13\,\hbox {mm}$$ and $$\hbox {height} = 10\,\hbox {mm}$$. The ceramic compound is designated by the manufacturer as SM410^36^ and presents the piezoelectric constants $$g_{31}$$ and $$g_{33}$$ equals to $$-10.3\times 10^{-3}$$ and $$23.3\times 10^{-3}\hbox {V}\,\hbox {m}/\hbox {N}$$ respectively, allowing to calculate the transducer sensitivity (Eq. 2), $$M_0 = 0.12\,\hbox {nV}/\upmu \hbox {Pa}$$ ($$-197.8\,\hbox {dB}$$ re $$1\hbox {V}/\upmu \hbox {Pa}$$).

#### Frequency range

The impedance of the transducer can give us valuable information. Impedance peaks and valleys indicate regions of resonance and antiresonance of the transducer, allowing to determine the portion of the frequency spectrum where the arriving signal will not be distorted^34^.

**Figure 2:**
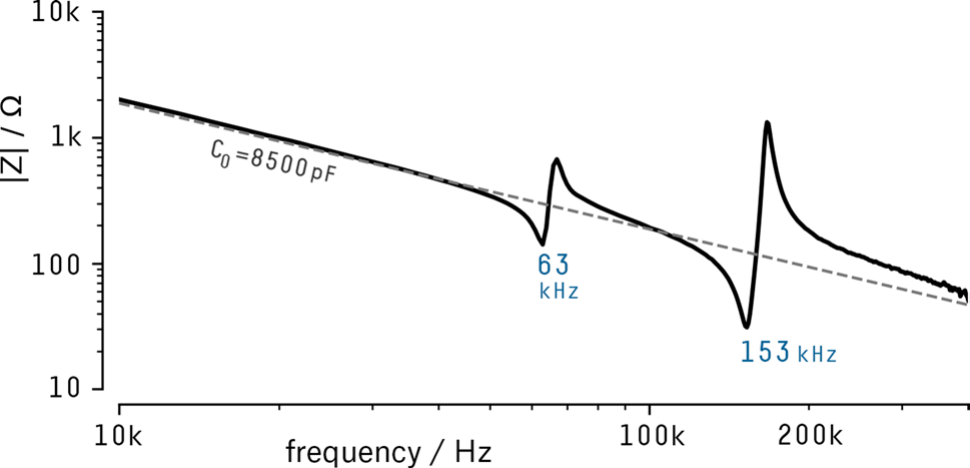
Piezo ceramic impedance magnitude focused on the higher portion of the spectrum where are the peaks of resonance and antiresonance. The dashed line represents a purely capacitive impedance of 8500 pF.

To measure the electrical properties of the transducer, a function generator (Agilent 33521B) was used to inject Gaussian pulses of broad spectral range ($\hbox {f}_{{\mathrm{max}}} \sim 500\,\hbox {kHz}$) on the capacitive divider created between a reference capacitor, $Z_{ref} = (2.50 \pm 0.25)\,\hbox {nF}$, and the piezoelectric ceramic. A digital oscilloscope (Agilent DSO-X 2012A) acquired the input and output signals, which were used to compute the complex impedance of the ceramic (Fig. 2).

It is possible to observe that in the lower frequencies, these ceramics behave like ideal capacitors (dashed line) until they reach the first region of resonance. The non-linear regions will set the operational upper limit of the sensor, and for the depicted ceramic $\hbox {f}_{\mathrm{max}} = 63\,\hbox {kHz}$, within the value range reported by the manufacturer specifications.

### Preamplifier

**Figure 3:**
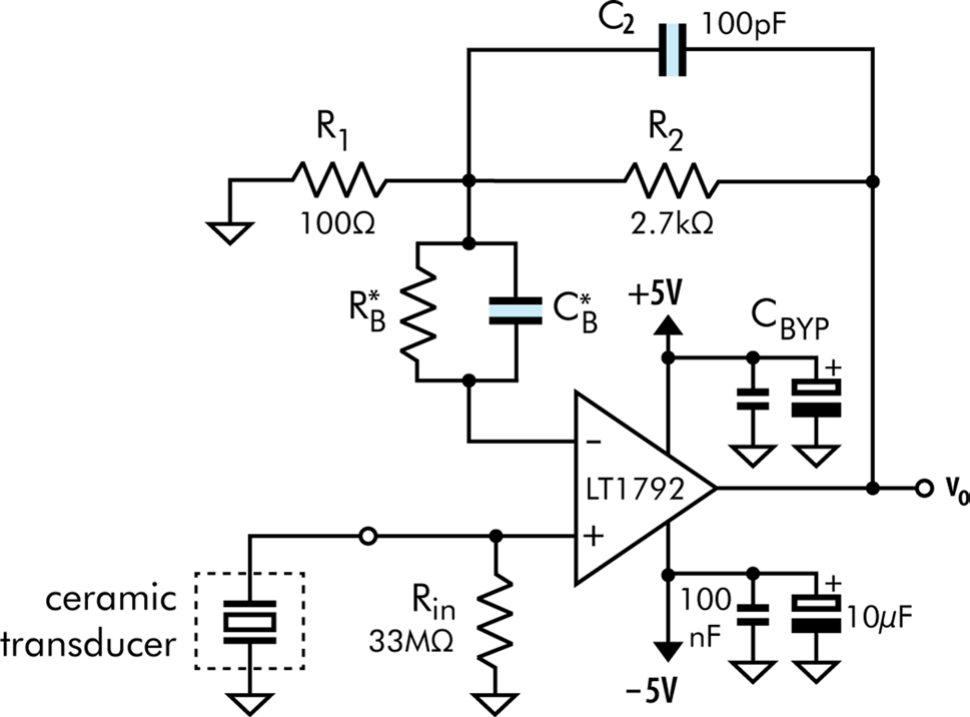
Low-noise preamplifier circuit. Parts $R_B$ and $C_B$ were not mounted, but they allow the possibility of future bias current compensations^37^.

Due to the low sensitivity and the high output impedance of the piezoelectric element (behaves as a capacitor in the lower portion of the spectrum), a preamplifier circuit was designed using an op-amp with FET inputs and wired in a non-inverting configuration (Fig. 3). The resistors $R_1$ and $R_2$ are used to set the amplification gain $G = 1+R_2/R_1$.

A monolithic op-amp was used at the heart of the preamp circuit. This chip needed to be carefully selected so as not to increase the sensor’s auto-noise and thereby impact the signal-to-noise ratio. Thus, the main parameters studied for choosing the integrated circuit were its noise characteristics and the impact of the ceramic transducer on the amplifier’s performance.

**Figure 4:**
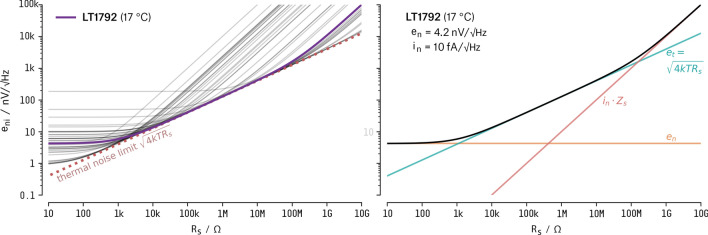
Noise PSD curves for a selection of 39 op-amps (left). Noise PSD for the selected op-amp, LT1792 (right).

A selection of 39 op-amps candidates, with promising noise characteristics was made for our application (for list of op-amps see supporting material). For each of these op-amps, the noise equivalent to input $e_{ni}$ (Fig. 4, left) was calculated using the op-amp noise model including source resistance $R_S$^38^.

The ideal preamp input impedance span is when the noise of the amplification electronics is less than the thermal noise of the transducer (i.e., a good candidate should have a noise curve very close to the minimum thermal noise). Based on the noise model values (Fig. 4, left), availability, and price, we chose the LT1792 (Linear Technologies) integrated circuit. The part has a broad range of excellent noise performance, allowing transducer impedances $R_S$ from $1\,\hbox {k}\Omega$ to $100\,\hbox {M}\Omega$ (Fig. 4, right). For values of RS below $1\,\hbox {k}\Omega$, the total noise related to the input $e_{ni}$ will be dominated by the thermal noise en of the op-amp (orange line). For $R_S$ values greater than $100\,\hbox {M}\Omega$, $e_{ni}$ will be ruled by op-amp current noise and will be equal to $i_n \cdot Z_S$.

The LT1792 (Linear Technologies) monolithic op-amp showed adequate specifications for the application (e.g.: $e_{n} = 4.2\,\hbox {nV}/\sqrt{\hbox {Hz}}$, $i_n = 10\,\hbox {fA}/\sqrt{\hbox {Hz}}$, $i_b = 800\,\hbox {fA}$, $\hbox {GBW} = 4\,\hbox {MHz}$) and relative low price ($\sim \$7.00/\hbox {un}$).

The final circuit utilizes metal-film resistor with values of $R_1 = 100\,\Omega$ and $R_2 = 2.7\,\hbox {k}\Omega$, yielding a gain of $\hbox {G} = 28 \,{\hbox {V}}/{\hbox {V}} = 28.9\,\hbox {dB}$.

The feed-back loop capacitor $C_2$ forms a low-pass filter with $R_2$ ($\hbox {f}_{{\mathrm{3dB}}} = 589\,\hbox {kHz}$), attenuating higher frequency signals and noise.

The resistor $R_{IN}$ serves to bleed accumulated charges on the transducer due to the small op-amp bias current $i_b$. Although one might think that its high resistance value would contribute with thermal noise ($v_n = \sqrt{4kBTR_{IN}}$)^39^, the combination of $R_{IN}$ with the transducer static capacitance ($C_0 \sim 8500\,\hbox {pF}$) forms a passive single-pole filter with a cutoff frequency around 0.57 Hz (to preserve low-frequency signals $R_{IN}$ should have a high value). When looking from the transducer side, the filter will have a high-pass behavior while from the resistor thermal noise source perspective, it will behave as a low-pass filter.

The sum of the transducer sensitivity, $M_0 = -197.8\,\hbox {dB}$, and preamp gain, $\hbox {G} = 28.9\,\hbox {dB}\,{\hbox {V}}/{\hbox {V}}$ equals to the total sensor sensitivity of $-168.9\,{\hbox {dB}}$ re $1\,\hbox {V}/\upmu \hbox {Pa}$.

#### Combined noise

**Figure 5:**
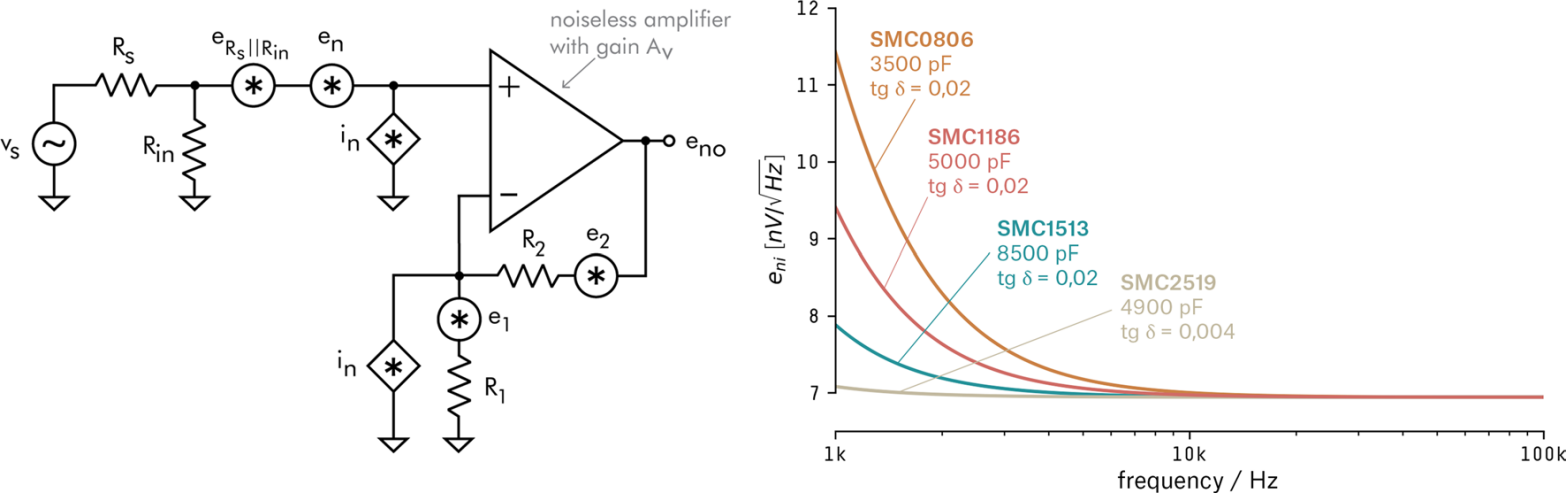
Final preamplifier noise model (left). Comparative analysis of the noise PSD for 4 different commercial piezo ceramic cylinders. Under the product name it is displayed the static capacitance of the cylinder $C_0$, and the dielectric loss factor $tg \, \delta$ (right).

Once defined the final preamplifier circuit, it is possible to do a more precise model of the total sensor noise, which accounts for the thermal noise of the transducer ($R_S$)^34, 40, 41^, the op-amp IC ($e_n$ and $i_n$) and the resistors, $R_1$, $R_2$, and $R_{IN}$ (Fig. 5, left). Since all sources are assumed independent, the addition of noise by each source can be calculated separately, and the results can be superposed^38^. The noise density relative to the input $e_{ni}$ can be calculated by^42^:

3 $$\begin{aligned} e^2_{ni} = e^2_n + e^2_{S || in} + \left[ i_n \cdot (R_S || R_{in}) \right] ^2 + e^2_{1 || 2} + \left[ i_n \cdot (R_1 || R_2) \right] ^2 \,\,\, [V^2{/}Hz]. \end{aligned}$$

The output result of the model for four different commercially available transducers shows a strong relationship between product’s intrinsic parameters, like capacitance and dielectric losses (Fig. 5, right).

The model prediction (Fig. 5, right) shows the noise power spectral density for the ceramic SMC1513 (blue curve), with a noise density of $e_{ni} < 8\,\hbox {nV}/\sqrt{\hbox {Hz}}$ for frequencies above 1 kHz (the SMC2519 ceramic was rejected due to its size and low resonance frequency). Consequently, the pressure equivalent noise power spectral density can also be calculated by $p_{ni} = e_{ni} / M_{0} = 63.5\,\upmu \hbox {Pa}/\sqrt{\hbox {Hz}} = 18\,{\hbox {dB}}$ re $\upmu \hbox {Pa}/\sqrt{\hbox {Hz}}$). For the majority of oceanic ambient-noise spectrum, the calculated noise is smaller than the minimum ocean noise (Wenz’s minimum) described in the work of Wenz^43^.

## Experimental setup

Experiments were performed using a non-anechoic tank with capacity for $170\,\textit{l}$ of water, $74 \times 56 \times 41\,\hbox {cm}^{3}$ (see Supplementary material for a field trial in a “bigger” tank). In all experiments, we used tap water in which temperature was monitored through a mercury-filled glass thermometer with markings every $1\,^{\circ }\hbox {C}$.

**Figure 6:**
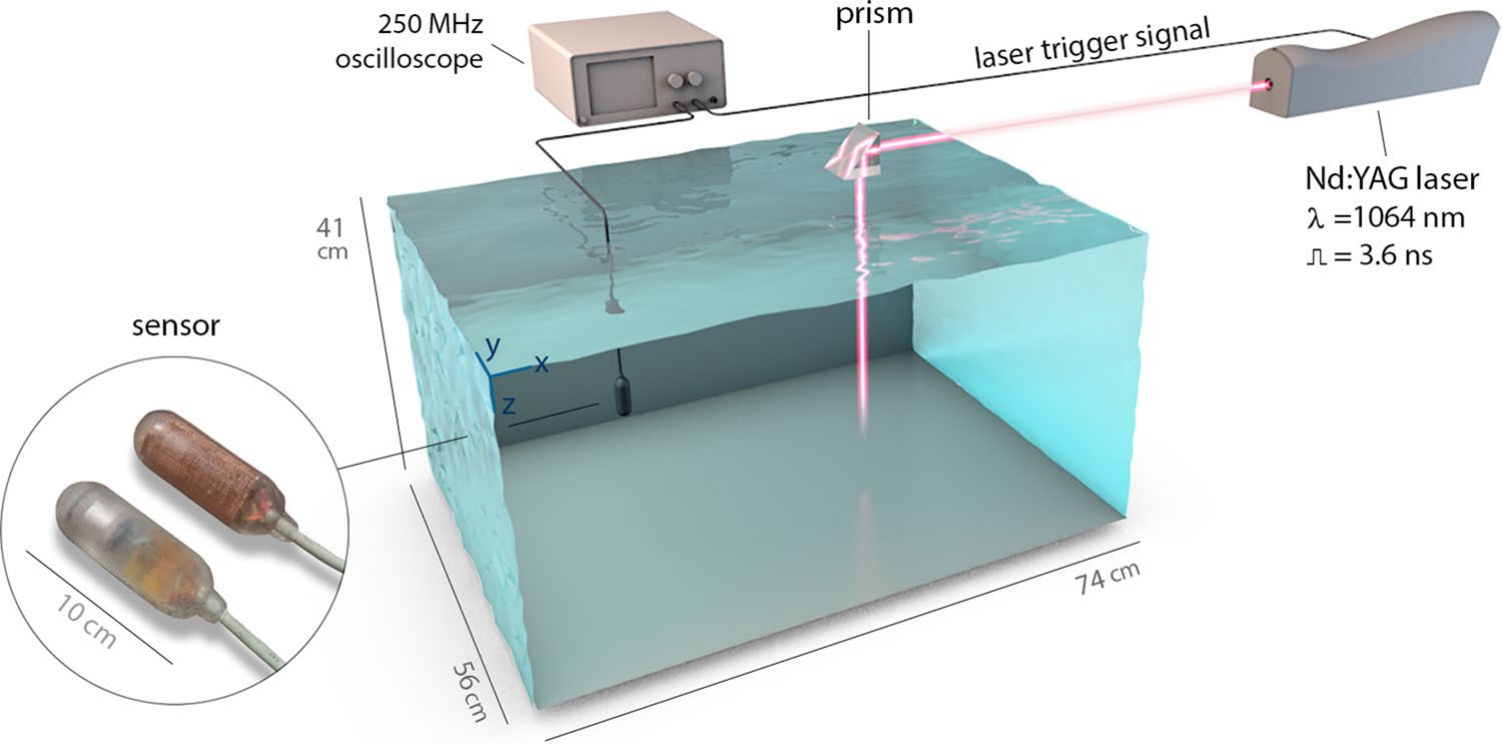
Simplified diagram of the experimental apparatus. Once the laser is commanded to fire, a trigger pulse is sent to the oscilloscope to allow the data capture synchronization. Then, all data is transferred to a computer. Inside the circle, a photograph of two thermoacoustic sensors.

As the source of pulsed energy, we used a Q-switched Nd:YAG laser (Quantel Brilliant-b) with a wavelength of $\lambda = 1064\,\hbox {nm}$, capable of generating pulses with adjustable energy up to 850 mJ (5.3 EeV), a fixed pulse width of 3.6 ns (FWHM), a spot size of 9 mm, and a Gaussian intensity profile. Since the laser was installed in an optical table, the beam was deflected to the liquid surface by means of a BK7 prism (Fig. 6).

Before the beginning of experiments, the adjusted pulse energy was verified using a pyroelectric sensor (Coherent J-50MB-YAG) with an active area diameter of 50 mm, an energy range of 1.5 mJ–3 J, a noise equivalent energy $<50\,\upmu \hbox {J}$, and calibration uncertainty of $\pm 2\%$. The detector was connected to a power and energy meter (Coherent LabMax-TOP) that allowed to measure pulses’ energy. The optical system uncertainty is considered to be lower than $\pm 5\%$.

The thermoacoustic sensor was mounted on a rigid rod with 3 degrees of freedom (x, y, z) over the whole tank volume. The origin of the adopted coordinate system was chosen to be the top corner indicated in Fig. 6.

The analog signal, generated by the sensor, was digitized through a 250 MHz oscilloscope (Tektronix MSO-4032) with a data buffer size of 100 000 samples, equivalent to a 0.4 ms acquisition window (4 ns interval between samples).

The experimental apparatus was automatized through a central computer running a Python script. The software controls the laser through an RS-232 interface, using the proprietary protocol defined by the laser manufacturer, and collects/stores the oscilloscope acquired signal using the VISA communication API.

## Results and discussion

### Acquired waveform

When firing the laser against the liquid surface, we are able to see a signal with different structures (shape, duration and temporal location). In order to be able to isolate the signal of interest—the one originated from the thermoacoustic phenomenon—it is important to understand the origin of each of these structures (e.g. thermodynamic, electromagnetic, mechanical).

**Figure 7:**
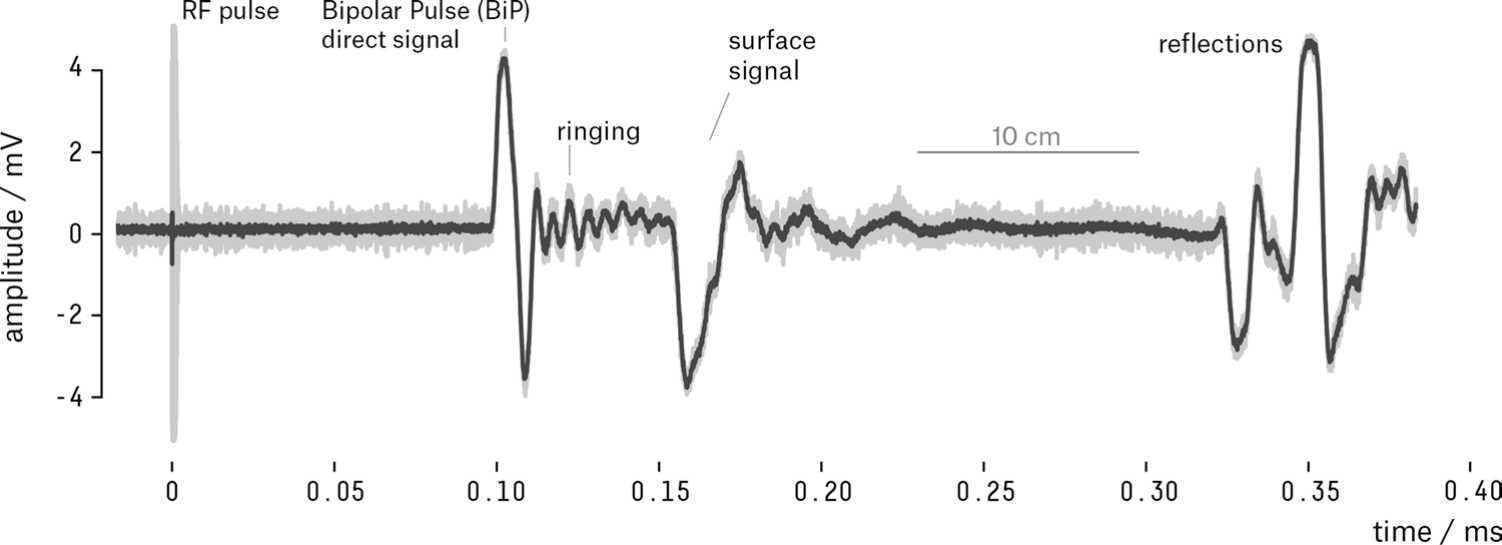
A sample waveform generated by the averaging of 256 laser pulses of 51.1 mJ acquired by the thermoacoustic sensor ($\hbox{T}_{\mathrm{H2}{\mathrm{O}}} = 19.9\,^{\circ }\hbox{C}$). The light grey curve depict the averaged waveform, and the dark grey the waveform filtered by a $4^{\mathrm{th}}$ order low-pass filter. The short horizontal line indicates the time necessary for the signal to travel a distance of 10 cm. The trigger interval of 0.1 s is long enough for all acoustic waves from the previous pulse to attenuate in the liquid medium.

Figure 7 shows an example of the characteristic waveform of the captured signals using the test tank with the thermoacoustic sensor positioned $(18.5 \pm 0.5)\,\hbox {cm}$ deep and at a horizontal distance of $(15.0 \pm 0.5)\,\hbox {cm}$ from the beam impact point on the water surface. The waveform is the average of a burst of 256 pulses ($f_{trigger} = 10\,\hbox {Hz}$) with an average energy and standard deviation of $(51.1 \pm 1.7)\,\hbox {mJ}$/pulse.

The acquired pressure signal is composed of some recurrent and distinguishable structures. The instant the laser beam is fired, $t = 0$, it is possible to see a sharp transient on the waveform. This signal could not have an acoustic origin because it was detected almost instantly with the laser trigger, and is the result of an RF pulse during the discharge of the capacitor bank of the laser source, as also observed in Hunter^44^. Despite being a “contamination” in the recorded signal, this pulse is valuable because it injects the instant the laser was fired into the data series.

About $100\,\upmu \hbox {s}$ after the impact of the laser beam on the free surface of the liquid, we can observe a bipolar pulse (BiP)—a compression pulse followed by a rarefaction pulse. This characteristic pulse is predicted by several studies^2–4, 45, 46^ as originated from the thermoelastic interaction process.

In the waveform, the bipolar signal is followed by a small amplitude oscillation attributed to the excitation of the resonance regions of the ceramic by the high-frequency components of the acoustic pressure signal, causing a ringing effect. The next pulse of remarkable amplitude comes from the surface, and it is the result of a significant energy deposition near the air/water interface where the transfer of the photons’ moment to the liquid medium generates a pressure pulse. This signal was also detected in other studies^32, 46^.

### Energy variation

**Figure 8:**
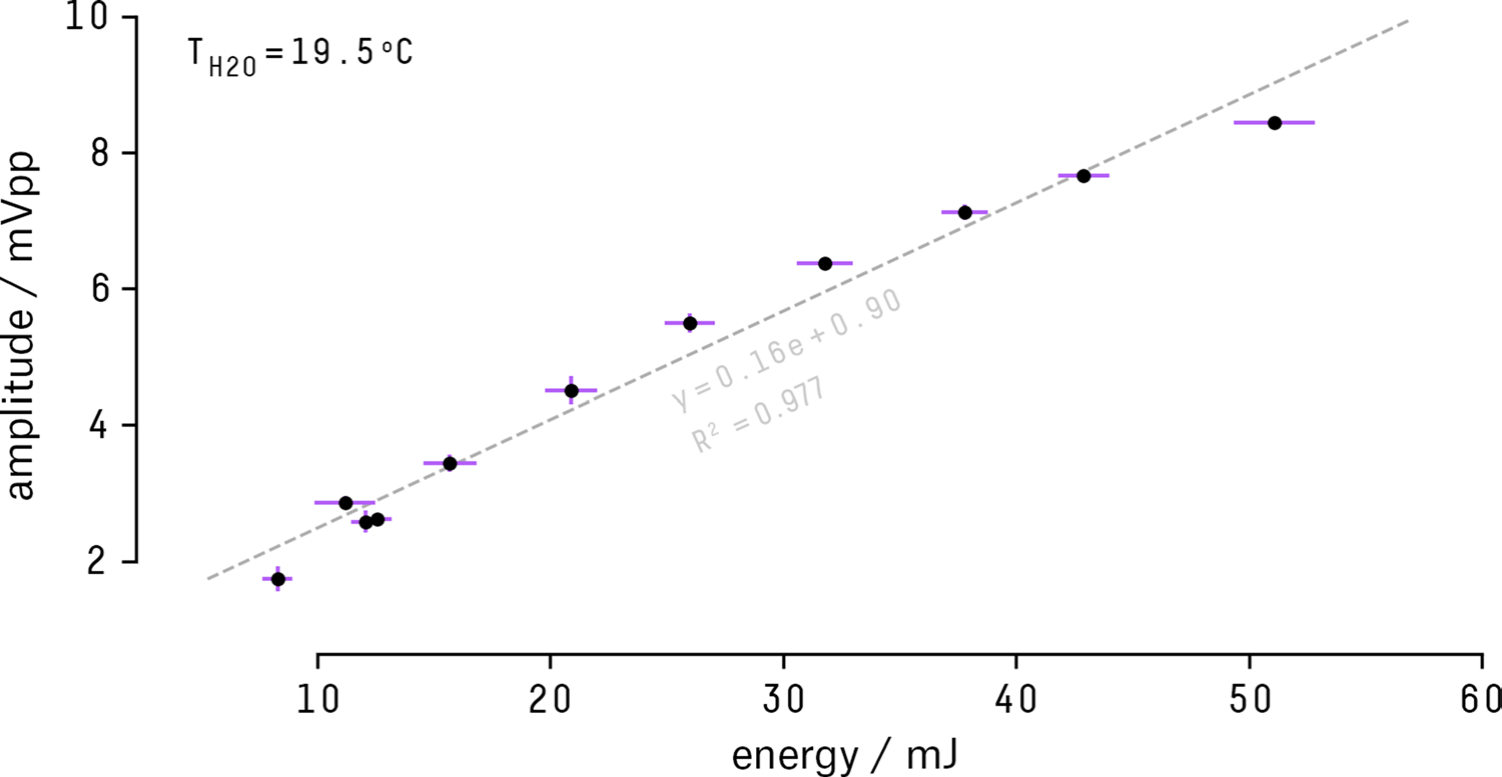
Acoustic signal amplitude variation (in volts peak-to-peak) with laser pulse energy (in mJ). The dashed line represents a linear regression between the bipolar signal amplitude measurements ($\textit{n}=2$) and pulse energy. The signal amplitude error bars denote the standard deviation of the mean amplitude from the two experiments. The energy error is the standard deviation of 256 pulses measured by the power meter.

In order to evaluate the variation of the pressure signal relative to the energy of the radiation pulse, the sensor was horizontally positioned $(15.0 \pm 0.5)\,\hbox {cm}$ away from the beam entrance. Two rounds of 11 measurements were made varying the pulse energy from 8.3 mJ to 51.1 mJ. On each run, the amplitude of the bipolar signal, from peak-to-peak, was calculated by filtering the average of 256 laser shots through a second order Butterworth low-pass filter. The result, displayed in Fig. 8, corresponds to the mean value between the two runs.

We observe a strong linear dependency between the signal amplitude and the total deposited energy ($\hbox {sig} = 0.16\hbox {e} + 0.90$), verified trough the coefficient of determination $R^2 \sim 0.977$. Although this result shows a promising trend, since the thermoacoustic model preaches a linear relationship between the two quantities^46, 47^, further experiments should be conducted to investigate the complete sensor response.

### Temperature variation

To test if the observed signals would change their polarity when the water temperature is below and above the point of maximum density of water, the following experiment was performed.

The thermoacoustic sensor was positioned at $(\hbox {x}, \hbox {y}, \hbox {z}) = (28.0, 28.5, 16.5)\,\hbox {cm}$. The beam contact point was adjusted to $(\hbox {x}, \hbox {y}) = (44.0, 31.5)\,\hbox {cm}$, resulting in a horizontal distance of 16.3 cm between the laser beam and the sensor. The tank was filled with equal amounts of water and ice. The experiment commenced only after the complete melting of the ice. The water temperature was left to increase gradually ($T_{amb} = 25\,^{\circ }\hbox {C}$), and measurements were performed at steps of approximately $0.5\,^{\circ }\hbox {C}$. Between each measurement, the water was hand steered in order to increase the homogeneity of the liquid volume.

For each target temperature, a burst of 256 laser pulses (10 Hz) with energy of 92.5 mJ/pulse was executed.

**Figure 9:**
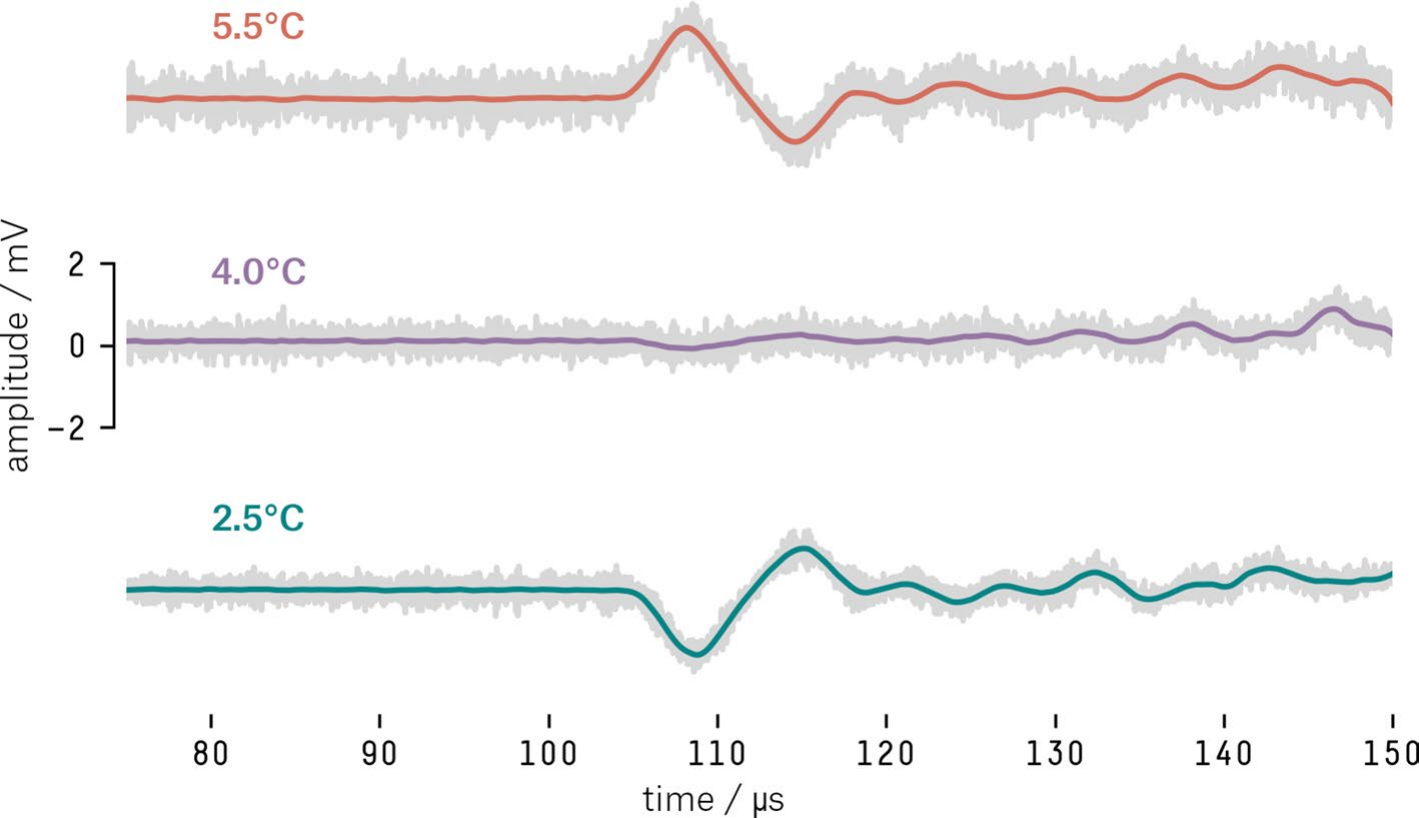
Inversion of the acoustic signal at three different temperatures (2.5, 4.0 and $5.5\,^{\circ }\hbox {C}$). The gray part corresponds to the average of 256 pulses and the colored curve the average after the application of a $2^{\mathrm{nd}}$ order Butterworth filter.

When the water was at $2.5\,^{\circ }\hbox {C}$ ($\beta _{\mathrm{H2}{\mathrm{O}}} < 0$), the first signal to arrive at the sensor was a rarefaction pulse followed by a compression pulse (Fig. 9, blue curve). As soon as the water temperature got closer to $4\,^{\circ }\hbox {C}$ (where $\beta_{\mathrm{H2}{\mathrm{O}}} \approx 0$), the signal vanished almost completely (Fig. 9, purple curve). And when the temperature was at $5.5\,^{\circ }{\hbox {C}}$ ($\beta _{\mathrm{H2}{\mathrm{O}}} > 0$), we observe an inversion of the bipolar pulse, first a compression pulse followed by a rarefaction one (Fig. 9, red curve).

In addition, we observed that the arrival time of the first peak occurred at $\hbox {t} = 108\,\upmu \hbox {s}$. Assuming the speed of sound in the water $c_s = 1426\,\hbox {m/s}$ at $5.5^{\circ }\hbox {C}$^48^, the signal source distance must be 15.4 cm away. As the calculated distance is approximately the distance between the sensor and the irradiated region, we can also presuppose the hydrodynamic origin of the signal.

## Conclusion

In this paper we presented the main steps on the development of a thermoacoustic sensor. Due to the experimental results discussed before (polarity inversion, bipolar shape, hydrodynamic origin, and linear relationship for energies up to $\sim 51\,\hbox {mJ}$), we are confident to state the thermoacoustic nature of the recorded signals. Hence, the proposed sensor design is a viable instrument (simple construction and low-cost) for experimental thermoacoustic research.

## Electronic supplementary material

Supplementary Information 1.

Supplementary Information 2.
