# Supplementary material for: Listening to pulses of radiation: design of a submersible thermoacoustic sensor
Source: Sci Rep. 2020 Jul 24;10:12433. doi: 10.1038/s41598-020-68954-8 (PMC7382503; doi:10.1038/s41598-020-68954-8)
Supplement: Supplementary file 1 — Supplementary Information 1. [file 41598_2020_68954_MOESM1_ESM.pdf]

Listening to pulses of radiation: design of a submersible thermoacoustic sensor

Rafael Barmak[1,*] **and Geraldo Cernicchiaro**[1]

[1]Brazilian Center for Research in Physics (CBPF), COMAN, Rio de Janeiro, 22290-180, Brazil.
*Corresponding author (rafabarmak@gmail.com)

Sea trials

One of the research objectives of this work was to make the sensor capable of withstanding the oceanic environment. The polyurethane (PU) resin that encapsulates the transducer and the preamplifier was chosen not only because it serves as a barrier against water but because of its acoustic compatibility (i.e. acoustic impedance) to water[1].

An exciting way to test the sensor's field capabilities (e.g., robustness) was to do a sea trial and attempt to detect a specific acoustic signal.

The most significant difficulty of this experiment was to find a sound source easily distinguishable among the countless sources of noise present in the ocean (e.g., sounds of biological origin, such as crustaceans in rocky walls, and anthropic sounds such as the passage of motorized boats). So we decided to use a pinger, a device used to locate submerged objects, from an aviation black-box scrap (more precisely, a flight data recorder – FDR). Once submerged, the pinger starts emitting a signal with a frequency of (37.5 ± 1.0) kHz, modulated by a square wave with a 1% duty-cycle (1 s interval, lasting 10 ms), and sound pressure level of 160.5 dB re 1 µPa at 1 m [2].

The test was carried out at Urca beach in Rio de Janeiro. At the north end of the beach, the pinger was installed (about 1 meter deep). At the south end, at a distance of 170 m, the thermoacoustic sensors were fixed (Figure B1). A 12 V SLA battery powered the sensor, and the output signal was captured using a portable audio recorder (Zoom H4n Pro) with 24-bit resolution and 96 kHz sampling rate[3].

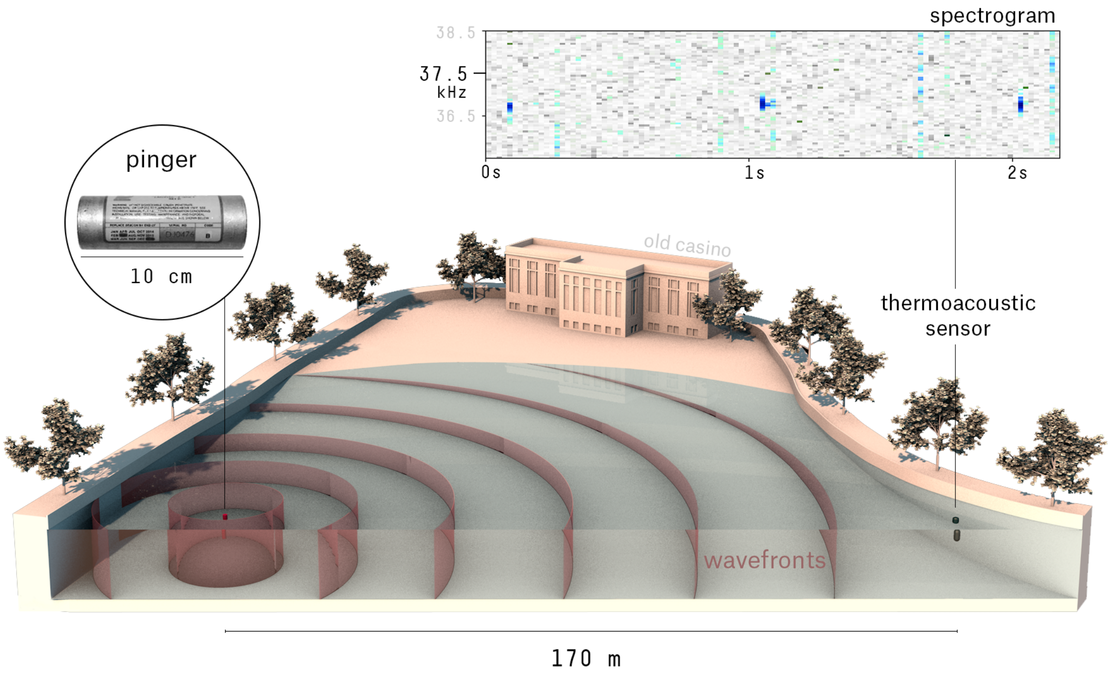

Figure 1. Illustration of the experiment location, at Urca beach in Rio de Janeiro (22°56′51.1″S, 43°9′49.3″W). The acoustic source (pinger) and the thermoacoustic sensor were installed 170 m apart. The red surfaces represent the acoustic wavefronts propagating away from the pinger (1 s intervals) until they reach the sensor. The top box is the spectrogram of a 2.2 s snippet of the sensor recording, clipped between 35.5 kHz and 38.5 kHz, when the pinger was active in the water. The blue dots, around 37 kHz, are points of high acoustic intensity and spacing close to 1 s.

The computed spectrogram for an audio snippet of 2.2 s from the recorded data (see supplementary material for the recorded audio file: `underwater_pinger_audio_10s.wav`) indicates intense pulses, when compared to the surrounding spectrum, centered at ∼37.0 kHz and intervals of ∼1 s (Figure 1, top box). Therefore, the pinger acoustic signal on the data stream was a positive indication of the TA sensor operational capabilities.

Detection range estimative

The flux of particles with ultra-high energies can be very low[4]. For instance, the flux of cosmic rays with energies of 10^{19} eV is $\sim$1/km^{2}year [5]. To better detect these rare events, it is important to understand the sensing range[6–10] of the thermoacoustic sensor developed in this work.

Once the energy from a cosmic particle is transferred to the water, the rapid expansion of the volume generates an acoustic pulse. This pulse will propagate through the water volume until it reaches a listening sensor or vanishes away. During the transit of an acoustic wave in the water, it will be attenuated mainly by the effects of divergence, or geometric spreading, and absorption, by molecular relaxation (in seawater strongly dominated by $MgSO_4$ and H_3BO_3)[11,12]. The work of Volovik *et al.* (1979)[13] presents an expression for the maximum pressure p_{max} (in Pa), at a distance r (in m), caused by a particle with energy $E = 10^{16}$ eV to 10^{21} eV, which takes into account the transmission loss,

$$p_{max}(r,E) = \frac{0.44 \cdot \phi(r)}{\sqrt{r}} \left(\frac{E}{10^{18}} \right). \quad (1)$$

The factor $\phi(r)$ adjusts the result for the deviation of a cylindrical spreading to the distance[14].

As previously discussed, the advantage of the acoustic method, over the optical, is its increased detection range. Neutrino oceanic observatories optical-modules are spaced in distances shorter than the light absorption length, that for blue light ($\lambda \sim$426 nm) it is $\sim$50 m to 55 m [15]. What would be the acoustic signal amplitude for a range ten times bigger than the blue light absorption length, $d = 500$ m? Using Eq. 1, the amplitude of a sonic signal generated by a particle with energy E = 10^{19} eV at a distance of 500 m (ϕ=0.28) will be 55.1 mPa.

The resulting electrical signal, found through the thermoacoustic sensor sensitivity (M_h = −168.9 dB re 1V/µPa = 3.59 nV/µPa), is equal to 197.8 µV. Now, this small captured signal can be processed by following amplifying stages until reaching appropriate levels for the Analog-to-Digital converter (ADC).

We must note that the ultimate sensitivity of the system will be dictated by the ambient noise level. Ocean noise is a complex subject, the following literature has a wide coverage on the topic:[12,16,17].

Temporal accuracy

We conducted an experiment to estimate the temporal accuracy of the sensor. Using a small acoustic tank, described in the Section "Experimental Setup", a waterproofed 35 mm piezoelectric disk was fixated at a depth of $h \sim 18.5$ cm, and excited by a signal generator (Agilent 33250A) with a burst of 5 cycles of a sinus at $f = 120$ kHz and amplitude of 10 Vpp.

The thermoacoustic sensor was installed in the extremity of a rigid rod attached to a robotic linear actuator (Daedal MD-2303), with a displacement resolution of 50 µm/step. The linear actuator was connected to a laptop able to issue moving commands. Before commencing the experiment, the sensor was placed 14.18 cm from the sound source, and its position was adjusted in 40 steps of 50 mm until reaching a total distance of 34.18 cm.

At each position, an average of 256 acoustic pulses, that traveled through the tank and reached the thermoacoustic sensor, was acquired through a digital oscilloscope with a sampling frequency of $f_s = 350$ MHz (Tektronix MSO-4034). The acoustic pulse propagation time was then calculated as the difference between the instant of signal injection on the piezoelectric disc and the time of arrival at the sensor.

Calculating the time difference between two consecutive measurements, we can have 40-time measurements over the same, precise, 50 mm distance. The resulting average propagation time is $\bar{t} = 3.39$ µs, and a standard-deviation $\sigma_t = 0.19$ µs, which can be considered as the temporal uncertainty of the device for this specific experimental setup. With the value of the sound-speed in water $c_s = 1504.6$ m/s ($T_w = 28.0$ °C)[18], the position uncertainty can be estimated through $\delta = c_s \cdot \sigma_t = 0.286$ mm (an error of $\sim$0.57% over the distance of 50 mm).

References

1. Cafarelli, A., Miloro, P., Verbeni, A., Carbone, M. & Menciassi, A. Speed of sound in rubber-based materials for ultrasonic phantoms. *J. Ultrasound* **19**, 251–256, DOI: 10.1007/s40477-016-0204-7 (2016).
2. Barmak, R. *et al.* Underwater Locator Beacon signal propagation on tropical waters. In *2017 IEEE/OES Acoustics in Underwater Geosciences Symposium (RIO Acoustics)*, 1–4, DOI: 10.1109/RIOAcoustics.2017.8349738 (2017).
3. Miyara, F. *et al.* Suitability of a consumer digital recorder for use in acoustical measurements. 9 (Lisbon, Portugal, 2010).
4. Particle Data Group *et al.* Review of Particle Physics. *Phys. Rev. D* **86**, 010001, DOI: 10.1103/PhysRevD.86.010001 (2012).

5. Nagano, M. Search for the end of the energy spectrum of primary cosmic rays. *New J. Phys.* **11**, 065012, DOI: 10.1088/1367-2630/11/6/065012 (2009).
6. Askariyan, G., Dolgoshein, B., Kalinovsky, A. & Mokhov, N. Acoustic detection of high energy particle showers in water. *Nucl. Instruments Methods* **164**, 267–278, DOI: 10.1016/0029-554X(79)90244-1 (1979).
7. Learned, J. G. Acoustic radiation by charged atomic particles in liquids: An analysis. *Phys. Rev. D* **19**, 3293–3307, DOI: 10.1103/PhysRevD.19.3293 (1979).
8. Lehtinen, N. G., Adam, S., Gratta, G., Berger, T. K. & Buckingham, M. J. Sensitivity of an underwater acoustic array to ultra-high energy neutrinos. *Astropart. Phys.* **17**, 279–292, DOI: 10.1016/S0927-6505(01)00158-X (2002).
9. Niess, V. & Bertin, V. Underwater acoustic detection of ultra high energy neutrinos. *Astropart. Phys.* **26**, 243–256, DOI: 10.1016/j.astropartphys.2006.06.005 (2006).
10. Bowen, T. Sonic particle detection. In *15th International Cosmic Ray Conference*, vol. 6, 277–282 (Budapest, 1977).
11. Bjørnø, L. *Applied Underwater Acoustics* (Elsevier, 2017).
12. Urick, R. J. *Principles of underwater sound* (McGraw-Hill New York, 1975).
13. Volovik, V. D., Kalinichenko, A. I., Lazurik, V. T. & Popov, G. F. The acoustic detection of high-energy particles in water. *Phys. Lett. A* **70**, 495–496, DOI: 10.1016/0375-9601(79)90377-3 (1979).
14. Lyamshev, L. M. *Radiation Acoustics* (CRC Press, 2004), 1st edn. p.328.
15. Capone, A. *et al.* Measurements of light transmission in deep sea with the AC9 transmissometer. *Nucl. Instruments Methods Phys. Res. Sect. A: Accel. Spectrometers, Detect. Assoc. Equip.* **487**, 423–434, DOI: 10.1016/S0168-9002(01)02194-5 (2002).
16. Wenz, G. M. Acoustic Ambient Noise in the Ocean: Spectra and Sources. *The J. Acoust. Soc. Am.* **34**, 1936–1956, DOI: 10.1121/1.1909155 (1962).
17. Mellen, R. H. The Thermal-Noise Limit in the Detection of Underwater Acoustic Signals. *The J. Acoust. Soc. Am.* **24**, 478–480, DOI: 10.1121/1.1906924 (1952).
18. Greenspan, M. & Tschiegg, C. Speed of sound in water by a direct method. *J. Res. Natl. Bureau Standards* **59**, 249, DOI: 10.6028/jres.059.028 (1957).
